# Improvement in Health Indicators of Islamic Republic of Iran in the Years 2004 and 2008

**Published:** 2011-08-01

**Authors:** S Goudarzi, M E kameli, H Hatami

**Affiliations:** 1 Health Network Improving Center, Ministry of Health and Medical Education, Shahid Beheshti University of Medical Sciences, Tehran, Iran; 2 School of Public Health, Shahid Beheshti University of Medical Sciences, Tehran, Iran

**Keywords:** Healthcare, Indicator, Performance evaluation, Medical university, Resource

## Abstract

**Background:**

As a performance evaluating program, healthcare indicators of the Islamic Republic of Iran at the end year of the 4th five-year socioeconomic strategic plan (2008) were evaluated in comparison with the same indicators at the 1st year of the 9th government (2004).

**Methods:**

The indicators were selected with the Delphi technique among the published indicators in the two period of time in 41 universities and in the country. Data gathering was done on the current health information system and were statistically analyzed assessing their trends.

**Results:**

The provinces of Sistan and Baluchistan (3.4%), Kerman (2.84%), Hormozgan (2.83%), Tehran (2.63%) and Qom (2.07%) had the highest rate of population growth over these years. Improving access to primary health care (PHC) in rural areas in Iran was evident during these years. The average hospital bed index in 1998 was one bed per 1000 population in the country and it was 1.62 in 2008. This Index was the highest in the province of Yazd and lowest in Ilam during both periods.

**Conclusion:**

A significant ascending trend was observed for indicators in all medical universities. A promotion in healthcare indicators in the lesser developed provinces seems necessary.

## Introduction

Although the overall level of health and related indicators were improved in the past decades but still there are many people who suffer from inequality of access to health services from economic, cultural and geographical aspects,[1] besides the health systems are still faced with numerous new challenges, including changes in diseases pattern and increasing noncommunicable and emerging diseases that threaten the health of people. On the other hand, the health systems were affected of economic crisis and lack of resources in the world and also with the advent of modern medical technology, the annual cost of many health systems and the people are increasing. According to the report of WHO in 2008, annually about 100 million people in the world are going under the line of poverty due to the health costs.[2] Broad health determinants have been used to inform public health interventions to improve individual and population health.[3]

During the past three decades with the formation of the network system in health and use of primary health care strategy, significant progress in health status is obtained in Islamic Republic of Iran. Among these achievements, the eradication of contagious diseases like smallpox and polio and a reduction in child and maternal mortality rates could be mentioned.[1]

Medical universities in Iran provide healthcare services to people in addition to carrying out their medical education and research. In approximately 95% of people who have access to primary healthcare,[4] this situation is related to some neighboring countries that were compared in Table 1. Determining the interval between the goals and current status is a critical issue in planning and policy making, so this study was done to evaluate the health indicators of medical universities as a performance evaluating program in Iran Ministry of Health and Medical Education.

**Table 1:** Health indicators of some WHO (EMRO) member countries [7]

**Indicators**	**Iran(2008)**	**Pakistan**	**Saudi Arabia**	**Egypt**	**Syrian Arab Republic**
Population growth rate (%)	1.6	1.7 e	2.2 e	2.1 d	2.5 e
Population with access to improved water source (%)	95.3	93 c	100 d	94 b	88 b
primary health care units and centers (per 10 000 population)	3.1	1.0 e	0.8 d	0.7 e	1.0 e
Hospital beds (per 10 000 population)	16.2	6.0 e	21.7 d	17.3 e	15.1 e
Population with access to local health services, rural (%)	95	100 d	…	90 e	100 d
Population with access to local health services, urban (%)	100	…	…	82 e	100 d
OPV3 %	100	98 d	91 d	86 e	94 d
Infant mortality rate (per 1000 live births)	27.0 a	70.2 c	17.4 d	17.0 d	15.5 b
Under five mortality rate (per 1000 live births)	19.9	90.0 c	21.1 d	21.8 d	22.0 b
Maternal mortality ratio (per 100000 live births)	24.1	27.6 c	14 d	55 d	58 b

^a^ 2005

^b^ 2006

^c^ 2007

^d^ 2008

^e^ 2009

## Material and Methods

A primary list of indicators by which we could have evaluated the input, process, outcome and impact of the health care system were selected by the Delphi technique from the current country registration system as mentioned above by a group of experts. Many indicators could not be changed during the period, so a short second list of indicators was selected. After gathering data at two cross sections including 2004 and 2008, related to the different universities, indicators that have predefined were estimated. The main indicators were as follows:

Population Growth Rate (GR), Access to primary health care (% of villages that have access to PHC), Access to hospital beds or Bed Index (bed per 1000 population), Access to family physician in rural regions, Bed occupancy (BO), Coverage of polio vaccine for children under 1 year, Death rate for children under 5 years per 1000 birth, Maternal mortality due to pregnancy complications(per 100,000 birth), Total physicians, Access to sanitary water in rural regions (% of villages that have access to sanitary water), Total number of ambulance per 1000 population, Rural health house per 1000 rural population, Urban health center per 12000 urban population and the first 10 main causes of death. Significant changes in the indices were studied in a trend study format and using paired T- test and the SPSS software.

## Results

The study shows that total urban population in Iran is increasing due to increasing trend of migration from neighbor countries and from internal rural regions. In most of the provinces, growth rate has an obviously decreasing trend however, it is due to the increased migration outside the province, except in Gilan Province (0.7%) that was due to a decrease in the birth rate.

The provinces of Sistan and Baluchistan (3.4%), Kerman (2.84%), Hormozgan (2.83%), Tehran (2.63%) and Qom (2.07%) had the highest rate of population growth over the years of 1996 to 2006. Of course, in Tehran (about 19% of the country population) and Qom, the high rate of population growth was mostly due to the increased migration from other provinces.

Improving access to primary health care in rural areas in Iran was evident during these years. Meanwhile, Zahedan University of Medical Sciences (in southeastern Iran) had the lowest index of villagers access to PHC both in 2004 and 2008 among the other medical universities, that was respectively 60% for 2004, and 77.5% in 2008. As can be seen in the Table 2, the average country index in 2004 was 95.43% and in 2008 was estimated 98.08%.

**Table 2:** The health indicators of Islamic Republic of Iran in the years; 2004 and 2008 [6]

	**Years**	****
	**2004**	**2008**
Population growth rate (%)	1.96	1.61
Access to PHC (%)	95.43	98.08
	sd:8.1	Sd:4.6
Access to family physician (%)	0^a^	96.87
		Sd:5.9
Bed index (per 1 000 population)	1^b^	1.62 [5]
Bed occupancy (%)	54^b^	66
Coverage of polio vaccine (%)	97.40	100
	sd:4.22	sd:3.7
Under five mortality rate (per 1000 live births)	24.37	19.93
	sd:7.44	sd:5.84
Maternal mortality ratio (per 100000 live births)	28.6	24.1
	sd:5.64	sd:6.7
Total physician	0.55	0.69
	sd:0.33	sd:0.38
Access to sanitary water (%)	86.16	90.5
	sd:13.7	sd:11.27
Total of ambulance (per 1 000 population)	2.7	4
Health house (per 1000 rural population)	0.73	0.76
Urban health center (per 12000 urban population)	0.62	0.63

^a^ Family physician program has started in 2004

^b^ In the year 1998.

Family physician program in Iran was actually started in 2004, so the related index was not available at the time but the country average access to family physician in rural regions was 96.87% in 2008. The average hospital bed index in 1998 was equal to one bed per 1000 population in the country and it was equivalent to 1.62 in 2008 (p=0.001). This Index was the highest in the province of Yazd and lowest in Ilam during both periods (1998 and 2008).

Polio vaccine coverage in rural population in Iran was 97.40 % in 2004 and 100% in 2008. The lower position belonged to Zahedan University of Medical Sciences in the year 2004 and Ilam University of Medical Sciences in southwestern of Iran in 2008.

The Highest levels of death rate for children under five years in the population of Birjand University of Medical Sciences from 44.46 (per 1000 live borne) in the year 2004 reached to 36.36 in Zahedan University of Medical Sciences with the country average 19.93 in the year 2008 in comparison to 24.37 in the year 2004 (p=0.001) amongst 41 universities of medical sciences. Eventually, the improvement occurred in the main indicators that are mentioned in Table 2.

**Fig. 1:**
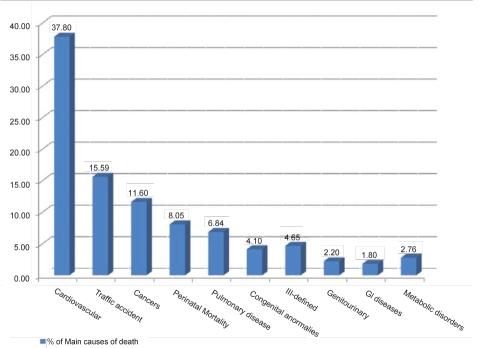
Frequencies of the first ten Main causes of death in IRI in the year 2008

Cardiovascular diseases with 37.80%, Traffic accidents with 15.59% and cancers with 11.6% (Figure 1) were frequencies of the first three causes of death for the population of Iran in the year 2008.

## Discussion

Although most of the main indicators in comparison with some neighboring countries (Table 1) in our study were significantly improved during these years, Iran healthcare performance evaluation of the health system was the main cause for conducting such a program in which the managers will be informed about their strengths and weaknesses and remind the policy makers in allocation of resources between the provinces. The lesser developed provinces in Iran with more population growth rate (e.g. Sistan and Baluchistan, Kerman and Hormozgan), have more problems in providing the primary and secondary healthcare services, so they should be considered as a high priority provinces for absorbing specialized manpower and extra financial resources from out of the provinces. On the other hand, there are few more developed provinces (e.g. Tehran) that have good position in the ranking of the indicators but they are confronting with the increasing marginal population growth rate, so the necessity of a faster intervention on providing primary healthcare services by intra provincial resource mobilization may be a more priority reaction.

Provinces with high hospital bed index (e.g. Yazd and Tehran) that inpatient facilities development had a more accelerated trend should be restricted in allocation of resources and other provinces with low bed index should be persuaded for establishing of new facilities.

Cardiovascular diseases, traffic accident, and cancers were first causes of death in 2008 in most provinces that shows more attention in disease control programs both in patient screening and life style improvement. Also the International Classification of Diseases (ICD) should be applied exactly by medial universities to establish more effective national death registry system and in the fields of lowering traffic accidents more cooperation between MOH and other stakeholders will be necessary. A significant ascending trend was observed for indicators in all medical universities. A promotion in healthcare indicators in the lesser developed provinces seems necessary.
